# Supplementary material for: The Essential Oil of Petroselinum crispum (Mill) Fuss Seeds from Peru: Phytotoxic Activity and In Silico Evaluation on the Target Enzyme of the Glyphosate Herbicide
Source: Plants (Basel). 2023 Jun 12;12(12):2288. doi: 10.3390/plants12122288 (PMC10305266; doi:10.3390/plants12122288)
Supplement: Supplementary file 1 [file plants-12-02288-s001.zip › plants-2364717-supplementary.pdf]

Supplementary material

The Essential Oil of *Petroselinum crispum* (Mill) Fuss Seeds from Peru: Phytotoxic Activity and In-Silico Evaluation on the Target Enzyme of the Glyphosate Herbicide

Oscar Herrera-Calderon [1,*], Abdulrahman M. Saleh [2], Ammar A. Razzak Mahmood [3], Mohamed A. Khalaf [4], James Calva [5], Eddie Loyola-Gonzales [6], Freddy Emilio Tataje-Napuri [7], Javier Hernan Chavez-Espinoza [8], José Santiago Almeida-Galindo [9], Haydee Chávez [8], and Josefa Bertha Pari-Olarte [8]

[1] Department of Pharmacology, Bromatology and Toxicology, Faculty of Pharmacy and Biochemistry, Universidad Nacional Mayor de San Marcos, Jr Puno 1002, Lima 15001, Peru.
[2] Pharmaceutical Medicinal Chemistry & Drug Design Department, Faculty of Pharmacy (Boys), Al-Azhar University, Cairo 11884, Egypt; abdo.saleh240@azhar.edu.eg
[3] College of Pharmacy- Department of Pharmaceutical Chemistry. University of Baghdad. Bab-Almouadam,10001, Baghdad, Iraq; kubbaammar1963@gmail.com
[4] Chemistry Department, College of Science, United Arab Emirates University, United Arab Emirates, 202170149@uaeu.ac.ae
[5] Departamento de Química, Universidad Técnica Particular de Loja, Loja 1101608, Ecuador; jwcalva@utpl.edu.ec
[6] Department of Pharmaceutical Science, Faculty of Pharmacy and Biochemistry, Universidad Nacional San Luis Gonzaga, Ica 11001, Peru; eddie.loyola@unica.edu.pe
[7] Departamento de Ciencias Comunitarias de la Facultad de Odontología, Universidad Nacional San Luis Gonzaga, Ica 11001, Peru; freddy.tataje@unica.edu.pe
[8] Department of Pharmaceutical Chemistry, Faculty of Pharmacy and Biochemistry, Universidad Nacional San Luis Gonzaga, Ica 11001, Peru; hchavez@unica.edu.pe (H.C.); bertha.pari@unica.edu.pe (J.B.P.-O.)
[9] Department of Basic Sciences, Faculty of Human Medicine, Universidad Nacional San Luis Gonzaga, Ica 11001, Peru; santiago.almeida@unica.edu.pe

* Correspondence: oherreraca@unmsm.edu.pe; Tel.: +51956550510

Table S1. Docking analysis of the volatile components of *P. Crispum* EO against EPSPS target.

**Targets screened**	**Tested compounds**	**RMSD value (Å)**	**Docking (Affinity) score (kcal/mol)**
EPSP synthase target site	Cubebol	1.80	-3.41
	trans-Carvyl acetate	1.54	-3.39
	Cryptone	1.69	-2.88
	Myristicin	1.42	-4.03
	cis-Piperitrol acetate	1.36	-3.54
	Glyphosate	0.19	-6.55
